# Analyses of the factors influencing the accuracy of three-dimensional ultrasound in comparison with cone-beam CT in image-guided radiotherapy for prostate cancer with or without pelvic lymph node irradiation

**DOI:** 10.1186/s13014-019-1217-0

**Published:** 2019-01-29

**Authors:** Sha Zhou, Liling Luo, Jibin Li, Maosheng Lin, Li Chen, Jianhui Shao, Shipei Lu, Yaru Ma, Yingting Zhang, Wenfen Chen, Mengzhong Liu, Shiliang Liu, Liru He

**Affiliations:** 1 Department of Radiation Oncology, Sun Yat-Sen University Cancer Center, State Key Laboratory of Oncology in South China, Collaborative Innovation Center for Cancer Medicine, No. 651, Dongfeng Road East, Guangzhou, 510060 China; 2 Department of Clinical Research, Sun Yat-Sen University Cancer Center, State Key Laboratory of Oncology in South China, Collaborative Innovation Center for Cancer Medicine, Guangzhou, China

**Keywords:** Prostate cancer, Three-dimensional ultrasound, Cone beam computed tomography, Image-guided radiotherapy

## Abstract

**Background:**

Three-dimensional ultrasound (3DUS) is an attractive option in image-guided radiotherapy (IGRT) for prostate cancer (PCa) patients. However, the potential factors influencing the accuracy of 3DUS in comparison with cone-beam CT (CBCT) in IGRT for PCa patients haven’t been clearly identified.

**Methods:**

The differences between US/US and CBCT/CT registrations were analyzed over 586 and 580 sessions for 24 and 25 PCa patients treated with or without pelvic lymph node irradiation, respectively. The clinical factors that may influence registration differences were also evaluated.

**Results:**

The average discrepancies between US/US and CBCT/CT registrations were − 0.28 ± 5.28 mm, − 0.16 ± 3.48 mm, and − 0.47 ± 4.31 mm in the superior-inferior (SI), left-right (LR), and anterior-posterior (AP) directions, respectively. The discrepancies were respectively less than 5 mm longitudinally, laterally, and vertically in 64.4 and 70.1%, 84.9 and 89.2%, and 75.9 and 79.1% of the patients treated with or without pelvic lymph node irradiation, respectively. The registration differences were significantly smaller at least in one direction in patients younger than 70 years, without pelvic lymph node irradiation, guided by transperineal ultrasonography and had a bladder volume smaller than 300 mL.

**Conclusions:**

Age, irradiated regions, 3DUS modality, and bladder volume are important factors that may influence the differences between US/US and CBCT/CT registrations. 3DUS guidance is more feasible for younger PCa patients with a better control of bladder volume during the treatment and those who did not undergo pelvic lymph node irradiation.

**Electronic supplementary material:**

The online version of this article (10.1186/s13014-019-1217-0) contains supplementary material, which is available to authorized users.

## Background

Image-guided radiotherapy (IGRT) is an essential prerequisite for accurate dose delivery in prostate cancer (PCa) radiotherapy [1–4]. Although cone-beam computed tomography (CBCT) is more widely used than invasive methods that use implanted surrogates for target localization, CBCT inevitably involves additional radiation exposure [4–6], which might contribute to the increased morbidity of secondary malignancies after radiotherapy [7]. In contrast, ultrasound (US) appears to be a more attractive option than the previously described methods since it allows real-time, volumetric, noninvasive, and nonionizing target tracking for patients [8].

The Clarity System (Elekta, Stockholm, Sweden) is one of the latest-generation US-based guidance systems that have been shown to be feasible for IGRT in PCa radiotherapy [9–14]. However, all of the published data using Clarity 3DUS focused on the primary treatment sites only (prostate or tumor bed post-prostatectomy), used relatively small sample sizes, and yielded inconsistent results [13, 15–19]. In addition, factors that may affect the accuracy of 3DUS in comparison with that of CBCT remain to be determined.

To date, no study has evaluated the feasibility of the 3DUS system for PCa patients treated with pelvic lymph node irradiation. However, pelvic lymph node irradiation is usually indicated for locally advanced PCa patients in China. Therefore, we conducted this study to explore the feasibility of using the Clarity 3DUS system for PCa patients treated with or without pelvic lymph node irradiation and to identify the relative influencing factors.

## Methods

### Patients and treatment course

This retrospective study evaluated the setup verification for 49 patients who received external beam radiation therapy for localized prostate cancer from June 2015 to February 2017 at Sun Yat-sen University Cancer Center. Thirty patients underwent definitive radiotherapy with a total dose of 67.5 Gy over 25 fractions, and the other 19 patients received adjuvant radiotherapy after prostatectomy with a total dose of 64–72 Gy over 32–36 fractions. Each patient was treated with a 6-MV linear accelerator (Elekta Versa HD; Elekta, Stockholm, Sweden) with a volumetric modulated arc therapy (VMAT) plan. Both 3DUS and CBCT scans were repeated before each treatment if possible after obtaining patient consent. This study was approved by the ethics committee of the Cancer Center of Sun Yat-sen University. Written informed consent was obtained from all the patients before treatment.

### Simulation

The workflow of Clarity was recommended by the manufacturer and was shown in Additional file 1: Figure S1. Daily quality control (QC) procedures were performed by physical technicians prior to scanning patients. All patients were asked to follow a protocol to ensure a filled bladder and an empty rectum before planning CT and each treatment session. In case of an empty bladder or flatulent rectum, patients were asked to drink water to fill the bladder or undergo an enema to empty the rectum [16]. Bladder volume and rectal volume were 409.32 ± 149.27 mL and 54.53 ± 25.50 mL during the course of the treatment, respectively. All patients were scanned in the supine position with a 3-mm slice thickness on a Big Bore CT scanner (Brilliance™ CT, Philips, The Netherlands). They were immobilized using a cushion under the knees to create a reproducible setup. The same position was kept immediately after CT acquisition to acquire the reference US image (US_ref_). For TAUS scanning, the US probe was manually placed 5–10 cm supra-pubic on the abdomen with a moderate pressure for a good quality of US images. Then the US probe is swept from superior to inferior, without translatory movement of the probe, to scan the prostate and bladder from retropubic to the top of bladder. For TPUS scanning, the transperineal US probe was placed on the perineum with a moderate pressure for good image quality, which allowed complete visualization of the penile bulb, prostate, and bladder. The US images were acquired using transabdominal ultrasonography (TAUS) for 30 patients undergoing definitive radiotherapy and using transperineal ultrasonography (TPUS) for the other 19 patients receiving adjuvant radiotherapy after prostatectomy.

### Position reference volume definition

All simulation CT images and information for all contours, including the gross tumor volume, clinical target volume (CTV), planning target volume (PTV), and organs at risk in Digital Imaging and Communications in Medicine (DICOM) format were imported into the workstation (Monaco 5.11.01; Elekta) as a reference for CBCT scans. The radiation treatment plan along with the CT images and all contour-related information were transferred into the Clarity planning workstation to define the reference positioning volume (RPV) on the US_ref_ image. For patients undergoing definitive radiotherapy, the RPV was the entire prostate. For those undergoing post-prostatectomy radiotherapy, the RPV corresponded to the bladder neck since it is included in the CTV according to the European Organization for Research on Treatment of Cancer (EORTC) guideline [20].

### Image acquisition and registration

Daily QC was performed using the Clarity quality assurance (QA) phantom by physical technicians, and all 3DUS and CBCT measurements were retrospectively revised by one senior physician (Additional file 2: Figure S2). Our results were in accordance with the previous report in which the errors are only about 1 mm radially [21].

For image guiding, all patients first underwent alignment according to skin landmarks. Patient repositioning was performed on the basis of CBCT/CT registration results. US images were collected for comparison purposes only in this study. A daily US image (US_daily_) was acquired before each treatment and registered on the US_ref_ image by manual movement in three orthogonal directions by a trained operator. CBCT images were acquired directly after the US_daily_/ US_ref_ registration.

The clip box for CBCT/CT registration was defined as the area which just covered the PTVs. In detail, for patients without pelvic lymph node irradiation, the clip box included the PTV of prostate ± seminal vesicle for definitive radiotherapy or tumor bed for postoperative radiotherapy; for patients with pelvic lymph node irradiation, the clip box included both the PTV of prostate ± seminal vesicle or tumor bed and the PTV of the pelvic lymphatic drainage area. We matched CBCT to the reference CT based on the whole pelvic and the prostate ± seminal vesicle or tumor bed for patients treated with and without pelvic lymph node radiotherapy, respectively. The registration was performed automatically based on grey values using Elekta software, followed by a manual adjustment by the operators on the soft-tissue target volume. No rotational setup errors were determined, and only translational shifts were considered. The times required for the acquisition and registration of US and CBCT were estimated to be 3 and 4 min, respectively.

### Data processing

A total of 1166 paired US and CBCT translational shifts were collected. For patient p and session s, setup errors were denoted as T_CBCT,*p*,*s*_ and T_US,*p*,*s*_ for the CBCT and US modalities, respectively. For each session, the difference between CBCT and US shifts was calculated in three orthogonal directions as follows: δ_CBCT-US,*p*,*s*_ = T_CBCT,*p*,*s*_ - T_US,*p*,*s*_. Means and standard deviations (SD) of the differences were calculated for all patients. To determine whether CBCT and US imaging had the same accuracy for prostate repositioning, the 95% limits of agreement (LOA) were calculated using the Bland–Altman method for each localization and each direction as follows: LOA = *b* ± 1.96*SD, where *b* is the bias (i.e., the mean of the differences between CBCT and US modalities), and SD is the standard deviation of the differences by assuming that the differences are normally distributed, indicating whether the difference between the upper and lower limits is acceptable in clinical practice [22]. Shift agreements, which were defined as the percentage of the number of sessions for which the difference between CBCT and US modalities was below 5 mm, were calculated.

The inter-operator variability (IOV) of the registration process for CBCT and 3DUS modalities was evaluated in each direction. A total of 40 images were selected for analysis. Twenty images of two patients undergoing lymph node irradiation and 20 images of two patients who did not undergo lymph node irradiation were retrospectively registered by two well-trained operators. For each session s of the patient p, the standard deviation σ_p,s_ was calculated over the assessments made by the two operators. The IOV was calculated using the following formula: IOV = RMS_*p*_(RMS_*s*_(σ_*p*,*s*_)), with RMS_*p*_ indicating the root mean square over all patients and RMS_*s*_ indicating the root mean square over all sessions for the same patients.

### Statistical analysis

All statistical analyses were performed using SPSS 20.0 (SPSS Inc., Chicago, IL), and *P* values < 0.05 were considered statistically significant. The shift differences between 3DUS and CBCT/CT registrations were compared using paired t-tests. The intergroup differences in categorical variables (i.e., those who underwent pelvic lymph node irradiation and those who did not) were compared using the chi-squared test. A logistic regression analysis was performed to identify significant factors associated with shift agreements [1: no (≥ ±5 mm) and 0: yes (< ±5 mm)], and odds ratios (ORs) and 95% confidence intervals were reported.

## Results

### Availability of comparisons

Forty-nine prostate cancer patients who underwent 1340 treatment sessions were included in this study. CBCT examinations were successfully performed in a total of 1331 sessions (99.33%) and 3DUS examinations were successfully performed in 1175 sessions (87.69%). Insufficient bladder filling was the most frequent obstacle for successful 3DUS. A total of 1166 paired US and CBCT translational shifts were calculated. All the following analyses are based on data from these 1166 paired shifts, and the accuracy evaluation of 3DUS was presented on the basis of the values provided by the soft-tissue match in CBCT.

### Comparison of setup errors in 3DUS versus CBCT

**Fig. 1:**
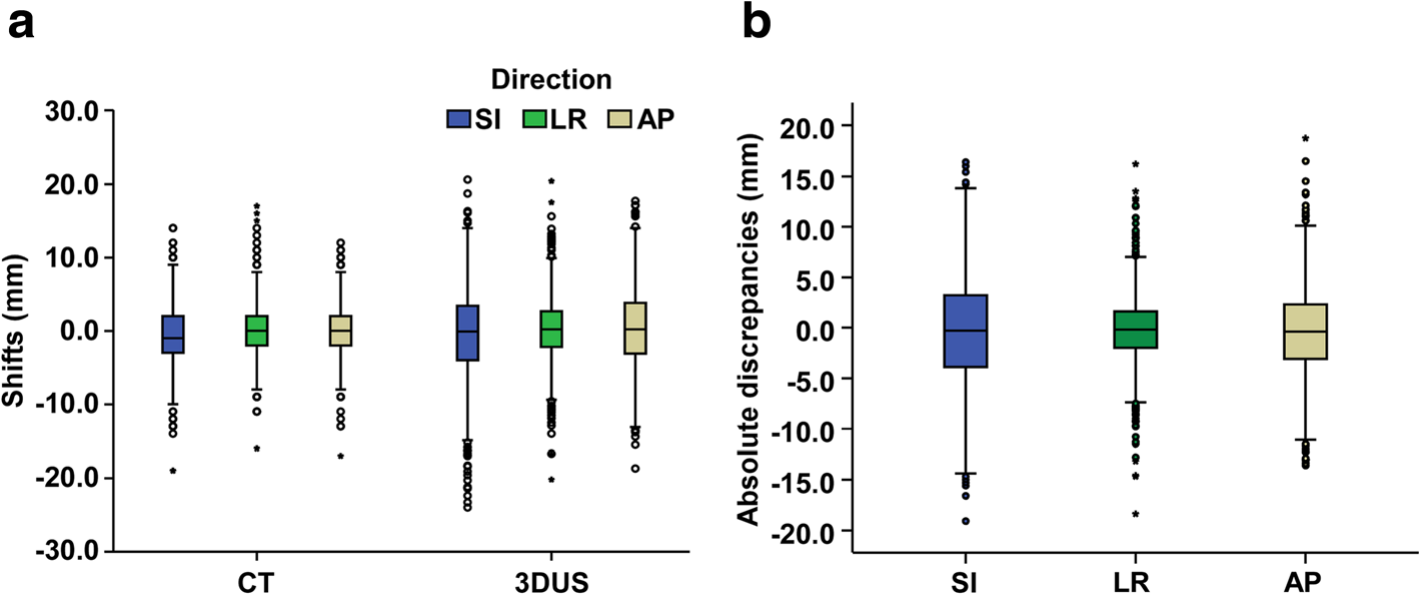
Box-and-whisker plots of prostate displacements. **a** Box-and-whisker plots of prostate displacements for all patients in the SI, LR, and AP directions for CBCT and US imaging, respectively. **b** The distribution of absolute discrepancies between 3DUS and CBCT shifts in the SI, LR, and AP directions. The box-and-whisker plots represent the median displacements observed during treatment courses (the horizontal band), the first (25th) and third (75th) quartiles (the lower and the upper edges of the box), and the total range (the lower and the upper extremes of the whiskers)

Setup errors in target localization between CBCT and the simulation CT were − 0.77 ± 3.98 mm, 0.05 ± 3.43 mm, and − 0.14 ± 3.33 mm in the SI, LR, and AP directions, respectively. For US imaging, setup errors were − 0.48 ± 6.09 mm, 0.23 ± 4.17 mm, and 0.40 ± 5.19 mm in the SI, LR, and AP directions, respectively (Fig. 1a). The average discrepancies between 3DUS and CBCT were − 0.28 ± 5.28 mm, − 0.16 ± 3.48 mm, and − 0.47 ± 4.31 mm in the SI, LR, and AP directions, respectively (Fig. 1b, Additional file 3: Table S1).

In evaluations using the Student’s two-sided one-sample t-test, the discrepancies between 3DUS and CBCT were not significantly different from zero in the SI (*P* = 0.066) or LR (*P* = 0.124) directions but showed significant differences in the AP direction (*P* < 0.001). Shift agreements for a range of ±5 mm were determined, and 67.6, 87.3, and 77.7% of the discrepancies were less than 5 mm longitudinally, laterally, and vertically, respectively. The upper and lower LOA values were 10.07 mm/− 10.63 mm, 6.66 mm/− 6.98 mm, and 7.98 mm/− 8.92 mm in the SI, LR, and AP directions, respectively (Additional file 3: Table S1).

### Comparison of the accuracy of 3DUS with reference to CBCT in patients treated with or without pelvic lymph node irradiation

**Fig. 2:**
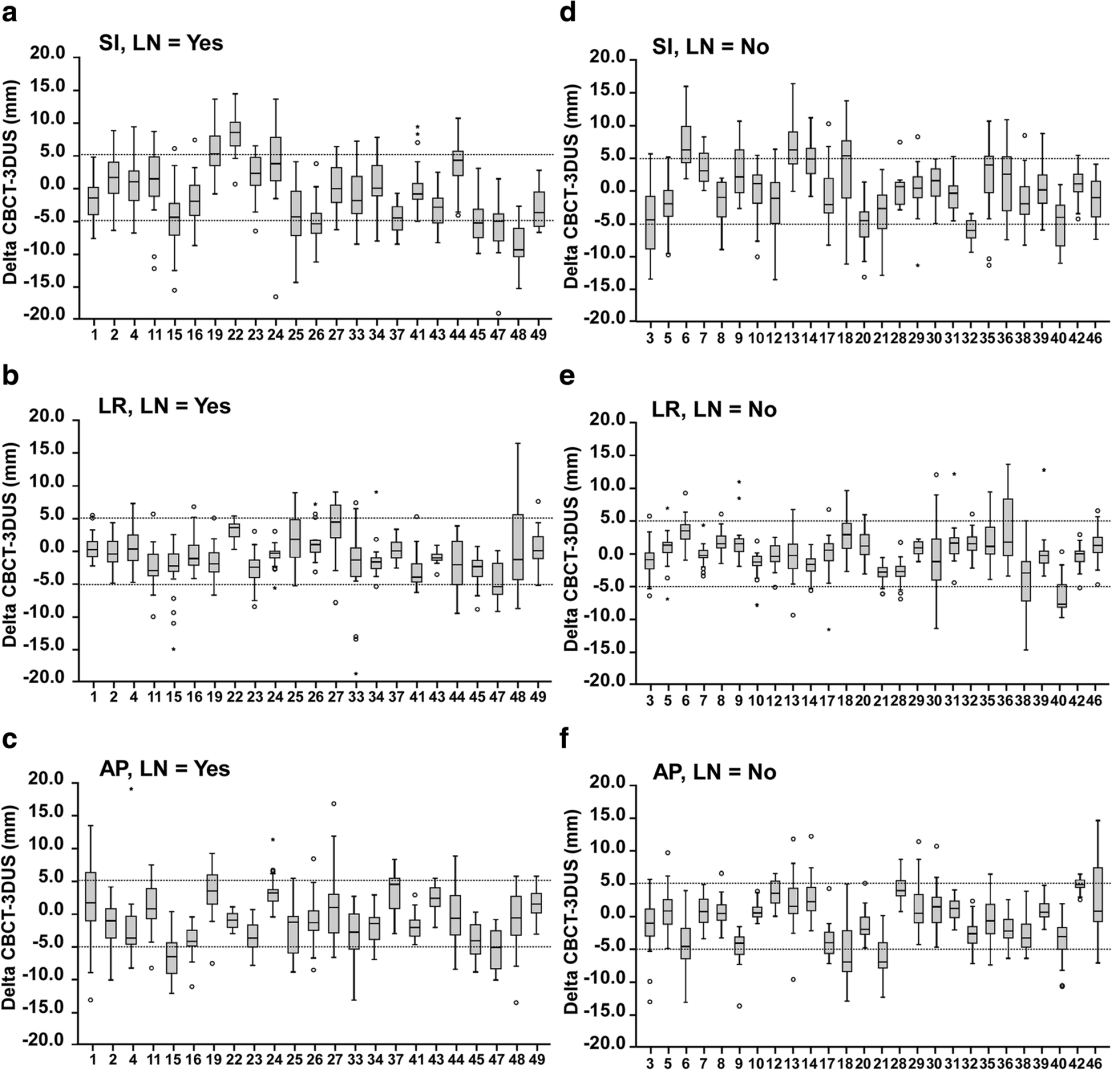
Discrepancies between 3DUS and CBCT shift from the raw dataset for each patient of groups A and B. **a**-**c** Discrepancies between 3DUS and CBCT shift for group A in the SI, LR, and AP directions. **d**-**f** Discrepancies between 3DUS and CBCT shift for group B in the SI, LR, and AP directions. Box-and-whisker plots represent the median discrepancies observed during treatment courses (the horizontal band), the first (25th) and third (75th) quartiles (the lower and the upper edges of the box), and the total range (the lower and the upper extremes of the whiskers). The dotted lines represent the ±5 mm range. Outliers are denoted by an asterisk

Patients were divided into two groups based on whether they received pelvic lymph node irradiation (group A) or not (group B). As shown in Fig. 2, the best concordance between the 3DUS and CBCT registrations was found in the LR direction, with 84.9 and 89.2% of the discrepancies being less than 5 mm in groups A and B, respectively. Larger absolute discrepancies between 3DUS and CBCT registrations were found in the SI and AP directions. The mean discrepancies between 3DUS and CBCT were significantly different in all the directions in group A (SI, *P* < 0.001; LR, *P* < 0.001; AP, *P* < 0.001) and in none of the directions in group B (SI, *P* = 0.143; LR, *P* = 0.153; AP, *P* = 0.227; Table 1).

**Table 1:** Comparison between CBCT and 3DUS shifts for group A and group B

	Group A (with pelvic lymph node irradiation)			Group B (without pelvic lymph node irradiation)		
	SI	LR	AP	SI	LR	AP
Mean ± SD	−1.03 ± 5.40	−0.60 ± 3.62	− 0.82 ± 4.41	0.29 ± 5.11	0.19 ± 3.33	− 0.20 ± 4.22
*P* value	< 0.001	< 0.001	< 0.001	0.143	0.153	0.227
Shift agreement (%)	64.4	84.9	75.9	70.1	89.2	79.1
LOA (mm)	(−11.61, 9.55)	(−7.70, 6.50)	(−9.46, 7.82)	(− 9.73, 10.31)	(− 6.34, 6.72)	(− 8.47, 8.07)

*CBCT* Cone-beam computed tomography, *3DUS* Three-dimensional ultrasound, *SI* Superior-inferior, *LR* Left-right, *AP* Anterior-posterior, *SD* Standard deviation, *LOA* Limits of agreement

### Comparison of the accuracy of 3DUS with reference to CBCT in patients undergoing TAUS and TPUS

**Fig. 3:**
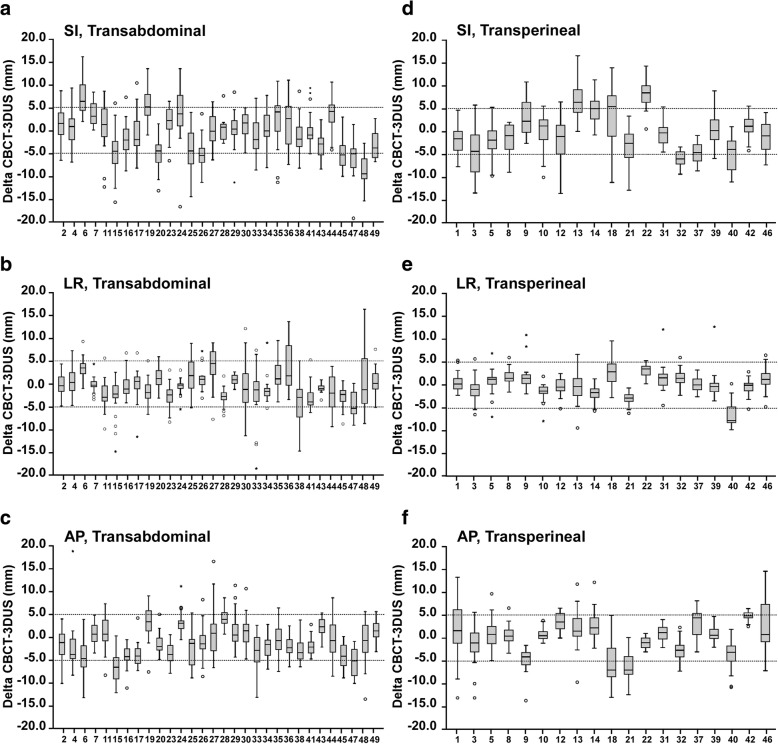
Discrepancies between 3DUS and CBCT shift from the raw dataset for each patient using the TAUS and TPUS modalities. **a**-**c** Discrepancies between 3DUS and CBCT shift for each patient using TAUS in the SI, LR, and AP directions. **d**-**f** Discrepancies between 3DUS and CBCT shift for each patient using TPUS in the SI, LR, and AP directions. Box-and-whisker plots represent the median discrepancies observed during treatment courses (the horizontal band), the first (25th) and third (75th) quartiles (the lower and the upper edges of the box), and the total range (the lower and the upper extremes of the whiskers). The dotted lines represent the ±5 mm range. Outliers are denoted by an asterisk

We investigated whether the relative accuracy of 3DUS with reference to CBCT is influenced by the approach used for acquiring US images. Patients were divided into TAUS and TPUS groups for further analysis. The best concordance between 3DUS and CBCT registrations was again noted in the LR direction, with 83.4 and 92.1% of the discrepancies being less than 5 mm in the TAUS and TPUS groups, respectively (Fig. 3, Table 2). In the other directions, all shift agreements were less than 80% and even under 70% in the SI direction (Fig. 3, Table 2). The discrepancies not significantly different in all directions in the TPUS group (SI, *P* = 0.899; LR, *P* = 0.151; AP, *P* = 0.103), but were significantly different in all directions in the TAUS group (SI, *P* = 0.019; LR, *P* = 0.005; AP, *P* < 0.001; Table 2).

**Table 2:** Comparison between CBCT and 3DUS shifts for patients using TAUS and TPUS modality

	TAUS			TPUS		
	SI	LR	AP	SI	LR	AP
Mean ± SD	−0.50 ± 5.32	− 0.44 ± 3.90	−1.11 ± 4.16	−0.03 ± 5.22	0.18 ± 2.87	0.31 ± 4.37
*P* value	0.019	0.005	< 0.001	0.899	0.151	0.103
Shift agreement (%)	67.8	83.4	78.5	67.3	92.1	76.7
LOA (mm)	(−10.93, 9.93)	(−8.08, 7.20)	(−9.26, 7.04)	(− 10.26, 10.20)	(−5.45, 5.81)	(− 8.26, 8.88)

*CBCT* Cone-beam computed tomography, *3DUS* Three-dimensional ultrasound, *TAUS* Transabdominal ultrasound, *TPUS* Transperineal ultrasound, *SI* Superior-inferior, *LR* Left-right, *AP* Anterior-posterior, *SD* Standard deviation, *LOA* Limits of agreement

### Factors that influence the accuracy of 3DUS with reference to CBCT findings

In order to identify the potential factors that influence the accuracy of 3DUS with reference to CBCT, the logistic regression analysis was performed. For the entire cohort, patients aged ≤ 70 years showed a significantly higher rate of shift agreement in all three directions than that shown by patients aged > 70 years (OR = 1.37, *P* = 0.012 for the SI direction; OR = 1.46, *P* = 0.036 for the LR direction; and OR = 1.70, *P* < 0.001 for the AP direction). However, the rectum volume showed no significant influence on the discrepancies between 3DUS and CBCT in any direction. Treatment without pelvic lymph node irradiation, bladder volume < 300 mL, and TPUS were significant influencing factors for the discrepancies between 3DUS and CBCT in the SI (OR = 1.30, *P* = 0.036) and LR directions (OR = 1.47, *P* = 0.03), in the LR (*P* = 0.004) and AP directions (*P* = 0.031), and in the LR direction (OR = 0.43, *P* < 0.001), respectively (Table 3).

**Table 3:** Analyses of factors that influence the accuracy of 3DUS to CBCT

	N	%	SI	*P*	%	LR	*P*	%	AP	*P*
			OR (95% CI)			OR (95% CI)			OR (95% CI)	
Age (years)										
≤ 70	21	28.7	1		10.5	1		17.5	1	
> 70	28	35.6	1.37 (1.07, 1.76)	0.012	14.6	1.46 (1.02, 2.08)	0.036	26.5	1.70 (1.28, 2.26)	< 0.001
Irradiated with pelvic lymph nodes										
No	27	29.9	1		10.8	1		20.9	1	
Yes	22	35.7	1.30 (1.02, 1.67)	0.036	15.1	1.47 (1.04, 2.07)	0.03	24.1	1.20 (0.91, 1.59)	0.189
3DUS modality										
TAUS	30	32.2	1		16.6	1		21.5	1	
TPUS	19	32.7	1.02 (0.80, 1.31)	0.85	7.9	0.43 (0.30, 0.63)	< 0.001	23.3	1.11 (0.84, 1.46)	0.476
Bladder volume (mL)				0.093			0.004			0.031
< 300	10	35.3	1		7.6	1		15.8	1	
300~399	11	29.3	0.76 (0.53, 1.10)	0.147	18.1	2.70 (1.56, 4.68)	< 0.001	24.5	1.73 (1.12, 2.66)	0.013
400~499	12	28.2	0.72 (0.51, 1.03)	0.07	11.7	1.62 (0.92, 2.87)	0.098	25.1	1.78 (1.17, 2.70)	0.007
≥ 500	16	35.9	1.03 (0.74, 1.43)	0.862	13.8	1.96 (1.14, 3.36)	0.015	23.6	1.64 (1.09, 2.46)	0.017
Rectum volume (mL)				0.498			0.42			0.079
< 40	18	34.2	1		14.2	1		20.5	1	
40~59.9	15	32.5	0.93 (0.69, 1.25)	0.618	12.6	0.87 (0.58, 1.31)	0.504	26.2	1.38 (0.99, 1.92)	0.055
≥ 60	16	30.3	0.84 (0.62, 1.13)	0.238	11.1	0.75 (0.49, 1.15)	0.189	20.3	0.99 (0.70, 1.40)	0.941

*CBCT* Cone-beam computed tomography, *3DUS* Three-dimensional ultrasound, *TAUS* Transabdominal ultrasound, *TPUS* Transperineal ultrasound, *SI* Superior-inferior, *LR* Left-right, *AP* Anterior-posterior, *OR* Odd ratio, *CI* Confidence interval

### The inter-operator variability

The IOV for both modalities is shown in Additional file 4: Table S2. The IOV values in the SI, LR, and AP directions were 1.4 mm, 1.3 mm, and 1.7 mm, respectively, for CBCT, and the corresponding values for US were 1.7 mm, 2.1 mm, and 2.6 mm.

## Discussion

3DUS guidance has definitively improved the accuracy of radiotherapy of prostate cancer. However, this image guidance modality still requires identification of its relative indicators. To date, most of the clinical experience was only related to prostate or tumor bed radiation. Little is known about the application of 3DUS to prostate patients treated with larger radiation area. To the best of the authors’ knowledge, the present study is the first to investigate the accuracy of 3DUS and the factors influencing its concordance with CBCT under clinical conditions with or without pelvic lymph node irradiation, in transperineal or transabdominal way.

Although there has been reported that the accuracy analysis of Clarity system using QA phantom was quite high (under ideal laboratory conditions, errors were about 1 mm radially) [21]. We were not surprised to find that the shift agreements of 3DUS to CBCT for the overall group were still low, especially in the SI (67.6%) and AP directions (77.7%). Some previous studies also have shown large discrepancies between the registration results of TAUS and CBCT, with shift agreements of only 73 to 77% and LOA values of about 10 mm in all directions [15, 23, 24]. Actually, our daily QC results were in accordance with the report from Ballhausen et al. [21]. However, we think it is reasonable for the difference between clinical usage and ideal lab conditions. Because the Clarity QA phantom is an ideal rigidity object, it does not move or transform during the image acquisitions and registrations. However, in clinical usage, the PRV often moves or transforms since the shape and volume of bladder and rectum may change during the treatments. Other factors, such as the different sizes between the clip boxes for CBCT and the US PRVs may also contribute to the relative large discordance between Clarity US and CBCT measurements in clinical usage.

We further compared the 3DUS and CBCT shifts in patients who underwent pelvic lymph node irradiation and those who did not, and found that the shift agreement was higher in all directions for those who did not undergo pelvic lymph node irradiation. The mean discrepancies were significantly different in all directions for patients who underwent pelvic lymph node irradiation but in none of the three directions for those who did not undergo pelvic lymph node irradiation. These data suggest that the irradiation area is an important factor influencing the accuracy of 3DUS with reference to CBCT for radiotherapy in prostate cancer.

In contrast of the large registration discrepancies between TAUS and CBCT mentioned above, Fargier-Voiron et al. reported a better shift agreement in comparisons of TPUS and CBCT, with values above 90% in all directions, except the AP direction (76.6%) for the prostate and the SI direction (85%) for post-prostatectomy cases [19]. In accordance with the previous studies, our results also showed that the mean discrepancies were significantly different in all directions in the TAUS group but showed no significant differences in any direction in the TPUS group. Several reasons may contribute to the differences between the TAUS and TPUS results. First, the quality of TAUS images is more reliant on a full bladder than that of TPUS images [25]. Fargier-Voiron et al. reported that 100% of the TPUS images and about 80% of the TAUS images had sufficient quality to be analyzed [19], which was similar to our experience. Second, variations in the probe pressure also affect the accuracy, and inter-operator uncertainties can be minimized by TPUS since the probe is fixed to a base plate and the sweeping is automated [26].

Interestingly, both age and bladder volume were identified as important factors that affect the accuracy of 3DUS with reference to CBCT. We suppose that the actual underlying factor is the repeatability of the bladder volume during the course of treatment. Although all patients were asked to hold a filled bladder in order to reduce the dose exposure to their bladder and intestine, patients younger than 70 years usually had better control of the bladder volume than the older patients. On the other hand, a bladder volume of less than 300 mL is easier for the PCa patients to repeat. Moreover, an excessively filled bladder may increase the urge to urinate and the risk of movement during the treatment. Taken together, our findings suggested that 3DUS is more feasible for PCa patients younger than 70 years with a filled bladder of no more than 300 mL.

Our study has some limitations: first, the impact of the probe pressure was not analyzed in our study since we did have any device to monitor the probe pressure during each treatment; second, since all US images for patients undergoing definitive radiotherapy were acquired by TAUS, and all the other US images for patients receiving adjuvant radiotherapy after prostatectomy were acquired by TPUS, we only considered the US modality but not the radiation purpose (definitive or adjuvant) in the analysis. Further studies that include more potential impact factors and larger patient populations are still needed to confirm our results.

## Conclusion

3DUS guidance is not safely interchangeable with CBCT for pre-treatment repositioning in all PCa patients. Age, irradiated regions, 3DUS modality, and bladder volume are important factors that may influence the accuracy of 3DUS with reference to CBCT in image-guided radiotherapy for prostate cancer. 3DUS guidance is more feasible for younger PCa patients with a better control of bladder volume during the treatments and those who are not receiving pelvic lymph node irradiation.

## Additional files

Additional file 1: **Figure S1.** The workflow of Clarity System recommended by the manufacturer. (TIF 703 kb)

Additional file 2: **Figure S2.** Daily quality control. Daily quality control data of the Clarity system from June 2015 to February 2017 at Sun Yat-sen University Cancer Center were shown. The Clarity™ calibration phantom was used to measure the discordance between Clarity and CBCT. (TIF 123 kb)

Additional file 3: **Table S1.** Comparison of setup errors in 3DUS versus CBCT for all patients in all three directions. (DOCX 18 kb)

Additional file 4: **Table S2.** Inter-operator variability of the registration of CT/CBCT and US/US images. (DOCX 15 kb)

## Abbreviations

3DUS: three-dimensional ultrasound
AP: Anterior-posterior
CBCT: Cone-beam computed tomography
CI: Confidence interval
CTV: Clinical target volume
DICOM: Digital Imaging and Communications in Medicine
EORTC: European Organization for Research on Treatment of Cancer
IGRT: Image-guided radiotherapy
IOV: Inter-operator variability
LOA: Limits of agreement
LR: Left-right
OR: Odd ratio
PCa: Prostate cancer
QA: Quality assurance
QC: Quality control
RPV: Reference positioning volume
SD: Standard deviations
SI: Superior-inferior
TAUS: Transabdominal ultrasound
TPUS: Transperineal ultrasound
US: Ultrasound
VMAT: Volumetric modulated arc therapy
