# Supplementary figures and images for: Analyses of the factors influencing the accuracy of three-dimensional ultrasound in comparison with cone-beam CT in image-guided radiotherapy for prostate cancer with or without pelvic lymph node irradiation
Source: Radiat Oncol. 2019 Jan 29;14:22. doi: 10.1186/s13014-019-1217-0 (PMC6352439; doi:10.1186/s13014-019-1217-0)

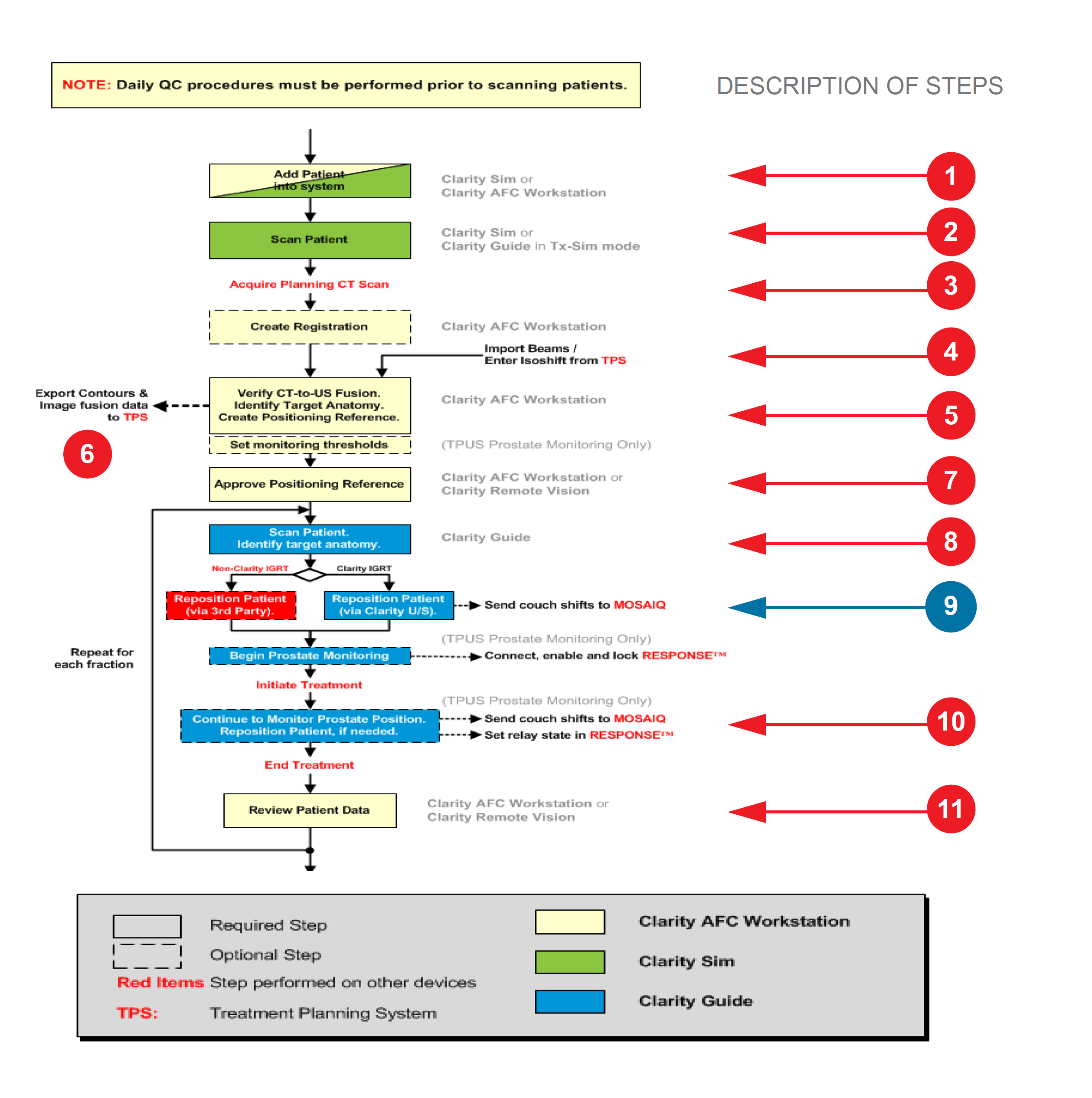

Supplement: Supplementary file 1 — Figure S1. The workflow of Clarity System recommended by the manufacturer. (TIF 703 kb) [file 13014_2019_1217_MOESM1_ESM.tif]

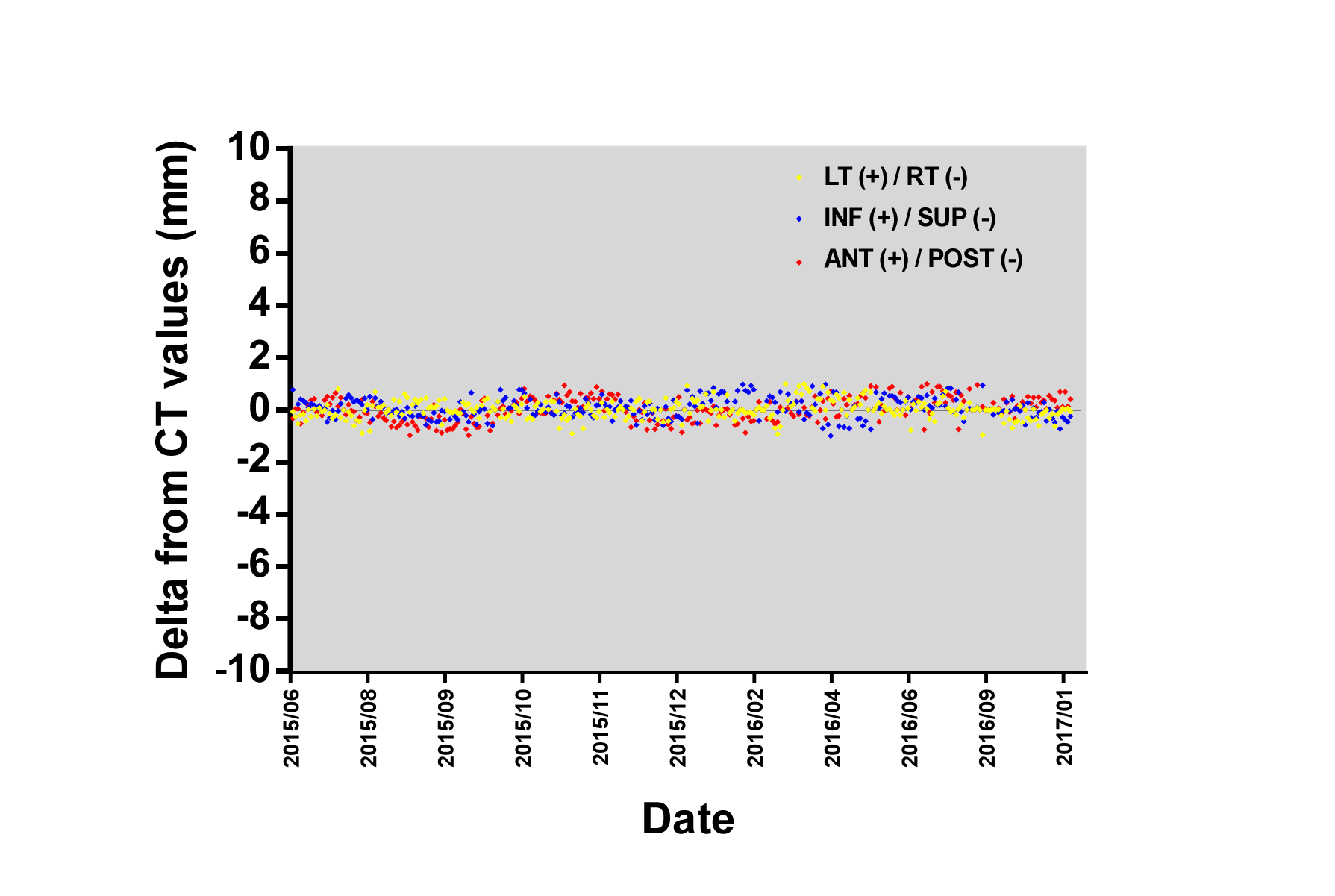

Supplement: Supplementary file 2 — Figure S2. Daily quality control. Daily quality control data of the Clarity system from June 2015 to February 2017 at Sun Yat-sen University Cancer Center were shown. The Clarity™ calibration phantom was used to measure the discordance between Clarity and CBCT. (TIF 123 kb) [file 13014_2019_1217_MOESM2_ESM.tif]
