# Supplementary material for: Analyses of the factors influencing the accuracy of three-dimensional ultrasound in comparison with cone-beam CT in image-guided radiotherapy for prostate cancer with or without pelvic lymph node irradiation
Source: Radiat Oncol. 2019 Jan 29;14:22. doi: 10.1186/s13014-019-1217-0 (PMC6352439; doi:10.1186/s13014-019-1217-0)
Supplement: Supplementary file 3 — Table S1. Comparison of setup errors in 3DUS versus CBCT for all patients in all three directions. (DOCX 18 kb) [file 13014_2019_1217_MOESM3_ESM.docx]

**Table S1.** Comparison of setup errors in 3DUS versus CBCT for all patients in all three directions.

|  | SI | LR | AP |
| --- | --- | --- | --- |
| Mean ± SD | -0.28 ± 5.28 | -0.16 ± 3.48 | -0.47 ± 4.31 |
| *P* value | 0.066 | 0.124 | <0.001 |
| Shift agreement (%) | 67.6 | 87.3 | 77.7 |
| LOA (mm) | (-10.63, 10.07) | (-6.98, 6.66) | (-8.92, 7.98) |

*CBCT* cone-beam computed tomography; *3DUS* three-dimensional ultrasound; *SI* superior-inferior; *LR* left-right; *AP* anterior-posterior; *SD* standard deviation; *LOA* limits of agreement.
