# Supplementary material for: Analyses of the factors influencing the accuracy of three-dimensional ultrasound in comparison with cone-beam CT in image-guided radiotherapy for prostate cancer with or without pelvic lymph node irradiation
Source: Radiat Oncol. 2019 Jan 29;14:22. doi: 10.1186/s13014-019-1217-0 (PMC6352439; doi:10.1186/s13014-019-1217-0)
Supplement: Supplementary file 4 — Table S2. Inter-operator variability of the registration of CT/CBCT and US/US images. (DOCX 15 kb) [file 13014_2019_1217_MOESM4_ESM.docx]

**Table S2.** Inter-operator variability of the registration of CT/CBCT and US/US images.

| (mm) | SI | LR | AP |
| --- | --- | --- | --- |
| CBCT | 1.4 | 1.3 | 1.7 |
| 3DUS | 1.7 | 2.1 | 2.6 |

*CBCT* cone-beam computed tomography; *3DUS* three-dimensional ultrasound; *SI* superior-inferior; *LR* left-right; *AP* anterior-posterior.
